# Idiopathic granulomatous mastitis masquerading as carcinoma of the breast: a case report and review of the literature

**DOI:** 10.1186/1477-7800-4-21

**Published:** 2007-07-27

**Authors:** Richard Tuli, Brian J O'Hara, Janet Hines, Anne L Rosenberg

**Affiliations:** 1 Dept. of Radiation Oncology, Johns Hopkins University School of Medicine, Baltimore, MD 21231, USA; 2 Dept. of Surgery, Thomas Jefferson University Hospital, Philadelphia, PA 19107, USA; 3 Infectious Diseases Division, Dept. of Medicine, Hospital of the University of Pennsylvania, Philadelphia, PA 19104, USA

## Abstract

**Background:**

Idiopathic granulomatous mastitis is an uncommon, benign entity with a diagnosis of exclusion. The typical clinical presentation of idiopathic granulomatous mastitis often mimics infection or malignancy. As a result, histopathological confirmation of idiopathic granulomatous mastitis combined with exclusion of infection, malignancy and other causes of granulomatous disease is absolutely necessary.

**Case Presentation:**

We present a case of a young woman with idiopathic granulomatous mastitis, initially mistaken for mastitis as well as breast carcinoma, and successfully treated with a course of corticosteroids.

**Conclusion:**

There is no clear clinical consensus regarding the ideal therapeutic management of idiopathic granulomatous mastitis. Treatment options include expectant management with spontaneous remission, corticosteroid therapy, immunosuppressive agents and extensive surgery for refractory cases.

## Background

Originally described by Kessler and Wolloch in 1972 [1], idiopathic granulomatous mastitis (IGM) is a rare disease of unknown etiology, which often mimics infection or malignancy and remains a diagnosis of exclusion. As a granulomatous form of lobular mastitis, IGM may be differentiated from granulomatous forms of periductal mastitis, as well as from granulomatous mastitis caused by sarcoidosis, Wegener's granulomatosis, giant cell arteritis, polyarteritis nodosum, foreign body reaction, and tuberculous, syphilitic, parasitic and mycotic infections [2,3]. Herein, we present a case of a young woman with IGM, initially mistaken for mastitis as well as breast carcinoma, and successfully treated with a course of corticosteroids.

## Case presentation

A 36 year-old pre-menopausal Asian woman previously in excellent health, presented with a three-month history of a progressively worsening tender right breast lump, with associated ulceration, induration, and erythema, draining serosanguinous fluid. She denied systemic symptoms, as well as any known recent fungal or tuberculosis exposure. The patient is a G2 P2002 who nursed her children without difficulty. She has had no history of breast disease, and her past medical and surgical histories are otherwise unremarkable. She denied tobacco use, any family history of breast diseases, use of any medications including oral contraceptives, or any known drug allergies. Physical examination was otherwise unremarkable with no palpable lymphadenopathy.

During initial presentation to an outside hospital, mammography and breast ultrasound revealed an increased density and ductal dilatation of the right breast with associated edematous changes. All laboratory and other radiological studies were otherwise normal. Although malignancy had not been excluded, the patient received a presumptive diagnosis of mastitis and was treated with a 7-day course of amoxicillin and cefadroxil without improvement. This was followed by a course of cefdinir and gatifloxacin, which was complicated by an antibiotic-induced erythema nodosum. As a result, antibiotics were discontinued and the patient was scheduled for an open breast biopsy. Interestingly, the patient's symptoms spontaneously resolved before tissue sampling was performed.

**Figure 1:**
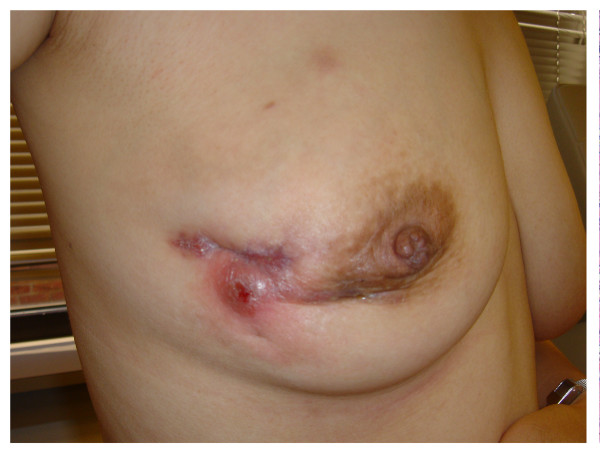
A patient with IGM of the right breast status-post incisional breast biopsy. Clinical presentation of IGM may mimic common entities such as breast mastitis, as well as more involved diagnoses such as malignancy.

**Figure 2:**
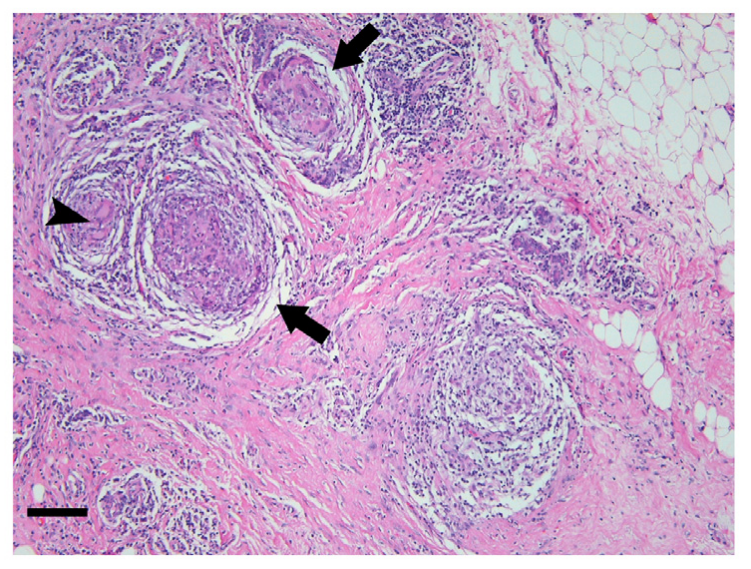
Photomicrograph of hematoxylin and eosin specimen from (1). Arrows indicate granulomatous inflammation centered on breast lobules, while arrowhead indicates the presence of multinucleated giant cells within non-caseating granulomatous inflammation (magnification 100×; bar = 150 μm).

Two months after her initial presentation, a second similar lump appeared in the right breast (Figure 1). A repeat ultrasound of this area again demonstrated dilated ducts and debris. Bilateral breast magnetic resonance imaging (MRI) revealed regionally dilated ducts with prominent regional ductal and parenchymal plateau enhancement. Given these findings were not typical for mastitis, and clinical and radiological data could not exclude breast carcinoma, tissue sampling was performed. Microscopic examination revealed chronic inflammation and macrophage, giant histiocyte and epithelioid-like cellular infiltration, with cytologic features suggestive of a granulomatous process (Figure 2). Further histopathological analysis showed no evidence of carcinoma or abscess formation, and all cultures and stains for infectious organisms remained negative. Secondary to exclusion of malignancy, infection, and other causes, the patient was given the presumptive diagnosis of idiopathic granulomatous mastitis (IGM). She was treated with a 6-month tapered course of prednisone with an excellent response, and has since remained free from recurrence.

## Discussion

Although the exact etiology of IGM remains unclear, associations with autoimmune disorders, oral contraceptive use, pregnancy, hyperprolactinemia and alpha-1-antitrypsin deficiency have been suggested [2,4-6]. Most studies report an average age of presentation in the third decade of life (range 11 to 83 years) with symptoms often developing within a few years of pregnancy [3]. Moreover, conflicting data exists regarding the significance of oral contraceptive use in patients diagnosed with IGM, with percentages of patients using contraception ranging from 0% to 33% [2,7,8]. However, no true associations with pregnancy, breast-feeding, prolactin levels, or oral contraceptive use have been established to date.

Interestingly, the majority of published reports of IGM have come from outside the U.S [9-13]. Whether this disproportional lack of published reports from the U.S. represents a lower prevalence of IGM, under diagnosis in the U.S., over diagnosis in developing countries, or a combination of the above remains to be addressed, yet underscores the necessity of appropriate diagnosis and treatment of IGM. Most frequently, the primary clinical finding is a unilateral firm breast mass affecting any quadrant of the breast, which may be tender in 25% of cases and present bilaterally in 25% of cases [2,3,14]. Patients with a more chronic presentation may go on to develop fistulae, abscesses, nipple inversion, and skin inflammation and ulceration over the course of several years [15]. In a recent study, Lai et al. reported that 100% of women with a histopathologically confirmed diagnosis of IGM initially presented with palpable breast masses [12]. Moreover, approximately 56% of women within this study were initially suspected to have breast carcinoma [12]. Given this initial presentation, combined with mammography, ultrasound and fine needle aspiration biopsy results that often mimic malignancy, several studies have documented the use of unnecessary mastectomies in patients with IGM [2,4,5]. Clearly, this underscores the importance of thorough histopathological analyses in patients with suspected breast carcinoma. A recent study assessed the potential utility of dynamic contrast-enhanced MRI in diagnosing IGM. Using patients with a histopathologically confirmed diagnosis of IGM, Kocaoglu et al. found varied appearances on MR and were unable to identify any imaging features characteristic of the disease, thereby suggesting its limited diagnostic utility [16].

The predominant and characteristic histopathological feature of IGM, granuloma formation, is also commonly found in other entities, thereby rendering it a diagnosis of exclusion. The lobular distribution of IGM represents a mixed chronic inflammatory process composed of lymphocytes, plasma cells, epithelioid histiocytes, multinucleated giant cells and less frequently, neutrophils [13,17]. However, confirmatory diagnosis is obtained only through identification of granulomatous inflammation centered on lobules (granulomatous lobulitis) with an absence of caseating necrosis. In more severe cases, confluency of the granulomatous inflammation may obliterate its typical lobulocentric distribution, thereby further complicating the diagnosis. Additionally, microabscess formation may occasionally involve the entire lobule, and squamous metaplasia of lobular and ductal epithelium may occur [3,18,19]. Although IGM patients rarely present with systemic signs of infection, culture specimens must nevertheless be analyzed for subtle microorganisms.

Likely secondary to the lack of published reports, particularly from the U.S., there is no clear clinical consensus regarding the ideal therapeutic management of IGM. Although several studies have reported varying approaches to the treatment of IGM, many of these treatment algorithms were formulated without a definitive initial diagnosis. Histopathological confirmation of IGM combined with exclusion of malignancy and other causes of granulomatous disease is of utmost importance in guiding clinical decision making and preventing inappropriate and unnecessary treatments. Therefore, following careful confirmation of diagnosis, the initial treatment for IGM clearly should be non-operative. Indeed, in a recent study, 50% of patients receiving expectant management had spontaneous and complete resolution of disease following a mean of 14.5 months [12]. An initial treatment option for patients with new-onset IGM with mild to moderate symptoms may be expectant management with close regular surveillance. For patients with clinically advanced disease or more severe symptoms, in whom infectious etiologies have been excluded, oral daily prednisone, starting with 0.8 mg/kg/day and tapering with clinical improvement, is a common regimen. Unfortunately, aside from the well-established side-effects of corticosteroid therapy, patients often relapse with cessation of therapy with one study reporting recurrence rates as high as 50% [11]. In cases of recurrent disease or those refractory to the above therapies, immunosuppressive agents, like methotrexate or azothioprine, have been utilized with variable responses [9,20], but minimal clinical evidence exists. Finally, surgical options should be explored in refractory cases or those with persistent collections. A retrospective study by Erhan et al. reviewed 18 women with clinicopathologically confirmed IGM treated with excisional biopsy [14]. According to the authors, recurrence was seen in only 3 women, 2 of whom were found to have hyperprolactinemia that was successfully treated with repeat excision and anti-prolactinemic therapy without subsequent recurrence. In cases with persistent abscess and fistula, wide surgical excision and even mastectomy may be indicated [2,10].

## Conclusion

IGM is a rare and benign inflammatory process commonly mistaken for malignancy and other disease entities. As a result, it is often incorrectly treated. It typically occurs in women during the third decade of life, presenting as an inflammatory palpable mass without systemic symptoms. Correct diagnosis requires the exclusion of infectious etiologies, other causes of granulomatous mastitis, and malignancy, combined with definitive histopathological confirmation. Treatment should initially be non-operative and depending upon the severity of initial presentation, may range from expectant management to corticosteroid therapy. In refractory cases, immunosuppressive agents and surgical excision may be indicated.

## Competing interests

The author(s) declare that they have no competing interests.

## Authors' contributions

RT performed chart review, literature search, and wrote and prepared the manuscript. BJO carried out histopathological analyses and review of the manuscript. JH treated the patient and reviewed the manuscript. ALR was the patient's surgeon and helped write and edit the manuscript. All authors have read and approved the final manuscript.
